# Imaging Evaluation of Periarticular Soft Tissue Masses in the Appendicular Skeleton: A Pictorial Review

**DOI:** 10.3390/jimaging11070217

**Published:** 2025-06-30

**Authors:** Francesco Pucciarelli, Maria Carla Faugno, Daniela Valanzuolo, Edoardo Massaro, Lorenzo Maria De Sanctis, Elisa Zaccaria, Marta Zerunian, Domenico De Santis, Michela Polici, Tiziano Polidori, Andrea Laghi, Damiano Caruso

**Affiliations:** 1 Radiology Unit, Department of Surgical and Medical Sciences and Translational Medicine, Sapienza University of Rome, Sant’Andrea Hospital, 00189 Rome, Italy; mariacarla.faugno@uniroma1.it (M.C.F.); daniela.valanzuolo@uniroma1.it (D.V.); massaro.1631569@studenti.uniroma1.it (E.M.); lorenzomaria.desanctis@uniroma1.it (L.M.D.S.); elisa.zaccaria@uniroma1.it (E.Z.); marta.zerunian@uniroma1.it (M.Z.); domenico.desantis@uniroma1.it (D.D.S.); michela.polici@uniroma1.it (M.P.); tiziano.polidori@uniroma1.it (T.P.); damiano.caruso@uniroma1.it (D.C.); 2 Department of Biomedical Sciences, Humanitas University, Pieve Emanuele, 20072 Milan, Italy; andrea.laghi@hunimed.eu.it; 3 Department of Diagnostic Imaging, IRCCS Humanitas Research Hospital, Rozzano, 20089 Milan, Italy

**Keywords:** soft tissue masses, joints, bursitis, lipoma, sarcoma, trauma, ultrasound, magnetic resonance imaging, computed tomography, clinical imaging, pathology

## Abstract

Soft tissue masses are predominantly benign, with a benign-to-malignant ratio exceeding 100:1, often located around joints. They may be contiguous or adjacent to joints or reflect systemic diseases or distant organ involvement. Clinically, they typically present as palpable swellings. Evaluation should consider duration, size, depth, and mobility. Also assess consistency, growth rate, symptoms, and history of trauma, infection, or malignancy. Laboratory tests are generally of limited diagnostic value. The primary clinical goal is to avoid unnecessary investigations or procedures for benign lesions while ensuring timely diagnosis and treatment of malignant ones. Imaging plays a central role: it confirms the presence of the lesion, assesses its location, size, and composition, differentiates between cystic and solid or benign and malignant features, and can sometimes provide a definitive diagnosis. Imaging is also crucial for biopsy planning, treatment strategy, identification of involved structures, and follow-up. Ultrasound (US) is the first-line imaging modality for palpable soft tissue masses due to its low cost, wide availability, and lack of ionizing radiation. If findings are inconclusive, magnetic resonance imaging (MRI) or computed tomography (CT) is recommended. This review aims to discuss the most common causes of periarticular soft tissue masses in the appendicular skeleton, focusing on clinical presentation and radiologic features.

## 1. Introduction

Soft tissue masses are a common clinical finding and are predominantly benign, with a benign-to-malignant ratio exceeding 100 to 1 [1]. These lesions typically arise around joints and can be directly associated with the joint capsule, synovium, or adjacent soft tissues. However, they may also be secondary to systemic diseases or reflect pathology in distant organs. Typically, they present as palpable bumps or swellings, and diagnostic evaluation should consider key clinical parameters such as duration, size, depth, mobility, consistency, growth rate, symptoms, and any history of trauma, inflammation, infection, or malignancy [2]. Soft tissue masses are particularly common in the appendicular skeleton. Ganglion cysts, for example, account for up to 30% of all wrist lesions and are typically observed in young adult females [Table 1]. Synovial cysts, lipomas, and bursitis are also frequently encountered and are often benign, self-limiting, and asymptomatic. Lipomas, one of the most common benign mesenchymal tumors, have an estimated incidence of 2.1 per 100,000 and usually affect middle-aged women. In contrast, osteochondromas tend to appear in younger males under the age of 20, while liposarcomas, though rare (0.5–1% of soft tissue tumors), require careful attention due to their malignant potential and insidious growth. Moreover, certain lesions, such as pigmented villonodular synovitis (PVNS) and giant cell tumors, although benign, may exhibit locally aggressive behavior and impair joint function over time (Table 1).

The main clinical challenge lies in distinguishing between benign and malignant or aggressive lesions early and accurately. This helps to avoid unwarranted interventions in benign cases while ensuring timely diagnosis and treatment for malignant ones [3]. In this context, imaging plays a pivotal role. It confirms the presence of a focal lesion, defines its size, location, and internal characteristics, and helps differentiate between cystic and solid or benign and malignant features. In selected cases, imaging alone may even be diagnostic. Moreover, imaging guides biopsy planning, treatment strategy, assessment of critical structure involvement, and follow-up [4]. Ultrasound (US) is the preferred first-line imaging modality for evaluating palpable soft tissue abnormalities [2]. According to the American College of Radiology (ACR), US is “usually appropriate” for superficial lesions and is increasingly used as the initial imaging modality for evaluating soft-tissue masses, particularly small superficial lesions located above the deep fascia. It is especially useful in suspected lipomas, which typically show no or minimal acoustic shadowing, low vascularity, and internal curved echogenic lines within a capsule. Hung et al. reported a sensitivity of 94.1% and specificity of 99.7% for US in the assessment of histologically confirmed superficial soft-tissue masses, with the highest accuracy for lipomas, followed by vascular malformations, epidermoid cysts, and nerve sheath tumors. Although US is highly accurate, further imaging is required when features are atypical, while MRI is recommended in cases of inconclusive US findings [5]. Compared to CT or MRI, US offers superior spatial resolution in superficial soft tissues and can detect submillimeter changes, whereas CT and MRI are more useful for deeper or larger lesions (≥5 mm). Moreover, MRI techniques such as Diffusion Weighted Imaging (DWI) can improve diagnostic accuracy both qualitatively and quantitatively, as demonstrated by several authors [6,7].

This review aims to provide a structured overview of the most common periarticular soft tissue masses involving the appendicular skeleton, describing their clinical presentation and imaging features. Lesions are grouped by etiology—traumatic, cystic, neoplastic, inflammatory, or degenerative—to facilitate a practical diagnostic approach.

## 2. Cystic-Fluid Lesions

Fluid soft tissue masses are very common around joints. X-rays are not useful in the diagnosis of fluid or cystic lesions; they may show soft tissue swelling or associated conditions like traumatic fracture or calcification [7]. In most cases the US allows the diagnosis: a typical fluid lesion appear as an anechoic, fluid-filled, distended structure, with a hyperechoic wall and a posterior acoustic enhancement [8,9] without internal Color-Doppler (CD) signals on US [10]. CD can play an important role in differentiating a small cyst from an aneurysm [11,12]. If present, synovial hypertrophy, hemorrhage, or loose bodies can be detected [13,14]; even communications with the articular cavity or other structures, in some cases can be detected by US [15]. On MRI, a fluid lesion shows the following signal characteristics: T2 hyperintensity and T1 hypointensity without fat suppression signal loss [16]. Additionally, MRI can reveal associated conditions such as tendon tears and assess deeper joints than US, also detecting synovial hypertrophy, hemorrhage, or loose bodies when present. CT is not typically the first line of imaging; when detected by CT, it typically shows a hypodense cystic lesion. An important role of CT is for guidance for procedures [17,18].

Ganglion cysts (GC) are fluid-filled sacs, the most common soft tissue masses in the hand and the wrist (representing 50% of all masses in these areas), and they are more common on the dorsal aspect of the wrist (60% to 70%) [8,9]. GC represents nearly 39% of soft tissue masses in the foot and ankle [19]. The presence of a communicating stalk with the joint or tendon sheath is typical of GC.

**Figure 1:**
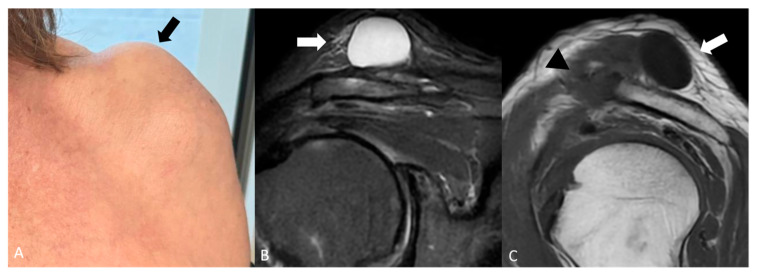
(**A**) Elderly woman with a soft bump on the cranial portion of her right shoulder (black arrow). (**B**) coronal STIR and (**C**) sagittal T1-weighted showing a cystic lesion superior to the acromion-clavicular joint (white arrows). The lesion is raising subcutaneous soft tissue. Acromioclavicular osteoarthritis is noted (black arrowhead).

A typical cystic shoulder bump is the “geyser sign” (GS) (Figure 1), a characteristic imaging finding associated with chronic rotator cuff tears and degenerative changes in the acromioclavicular (AC) joint capsule. This sign is visualized as a fluid-filled collection above the AC joint, extending from the subacromial bursa through the compromised AC joint capsule into a subcutaneous space above the clavicle [16].

The most common cystic lesion around the knee joint is the Baker’s cyst (BC) or popliteal synovial cyst: a fluid-filled sac that forms in the popliteal fossa (the shallow depression located at the back of the knee). It is usually located posteriorly or posterolaterally, between the medial head of the gastrocnemius and the semimembranosus; lateral localization is rare [20]. BC are classified as primary or secondary, depending on whether there is communication with the knee joint (secondary) or not (primary) [15,20,21].

**Figure 2:**
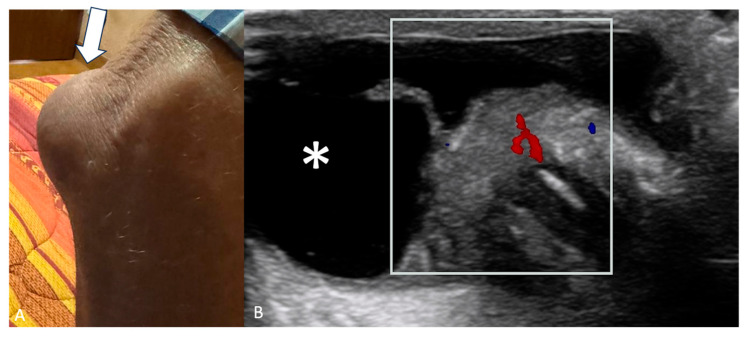
(**A**) Right elbow of a 73-year-old male with a bump in the posterior region of the elbow (white arrow). (**B**) US shows anechoic fluid collection (asterisk) with thickened and hypervascular synovium (white box) overlying the olecranon process of the ulna, compatible with olecranon bursitis.

Bursitis may appear as a soft tissue mass around joints. Subacromial-subdeltoid (SASD) bursitis is the most common of the shoulder [12], olecranon bursitis (OB) (Figure 2) is the inflammation of the olecranon bursa, a fluid-filled sac overlying the olecranon process of the elbow [22], and trochanteric bursitis (TB) is a common cause of lateral hip pain due to inflammation of the trochanteric [23]. Increased vascularity on CD can aid in assessing active inflammation associated with bursitis but is not so common [17,18].

**Figure 3:**
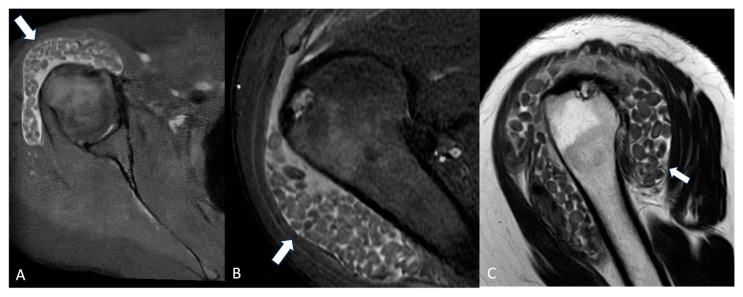
Axial PD-FS (**A**), coronal PD-FS (**B**), and T2-weighted (**C**) MRI images of a 48-year-old female patient presenting with shoulder pain and swelling. Multiple non-osseous cartilaginous loose bodies (arrows) are visible within the bursa, which appears enlarged, causing bulging of the deltoid muscle. Findings are consistent with synovial chondromatosis.

Synovial chondromatosis (Figure 3) may develop as a complication of chronic bursitis [24].

## 3. Fatty Lesions

**Figure 4:**
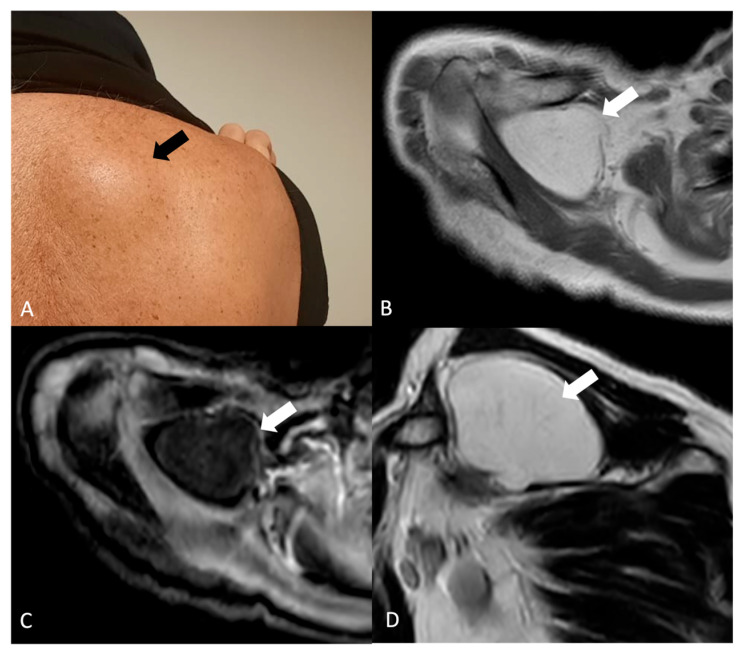
(**A**) Right shoulder bump of a 67-year-old male patient (black arrow). (**B**) Axial T1-weighted, (**C**) axial T1-weighted-fs and (**D**) sagittal T2w show a fat-containing (white arrow) lesion. No internal septations or nodular components are observed. The imaging features and typical superficial location are conclusive for a benign lipoma.

Lipomas (Figure 4) are benign tumors arising from adipose tissue and are the most common soft tissue tumors in adults and are generally more frequent in middle-aged and older adults, with a slight male predominance reported in some studies. Lipomas can manifest with a spectrum of clinical presentations depending on their size, location, and compression of adjacent structures (impingement syndrome, limited range of motion, and pain) [25].

On US, lipomas appear as well-defined, hyperechoic lesions relative to surrounding muscle with a characteristic homogeneous internal structure. US may also be useful for guiding fine-needle aspiration biopsy for further evaluation [25]. On MRI, lipomas typically appear as well-circumscribed, high-signal intensity lesions on T1-weighted images with corresponding signal suppression on fat-suppressed sequences [25]. MRI can be particularly helpful in evaluating the extent of the lipoma and its relationship to surrounding neurovascular structures. On CT, lipomas appear as circumscribed, low-attenuation masses (typically approximately −65 to −120 HU) with minimal internal soft-tissue components. Areas of calcification may be present, although they are more frequently associated with well-differentiated liposarcoma [25].

## 4. Bony Lesions

**Figure 5:**
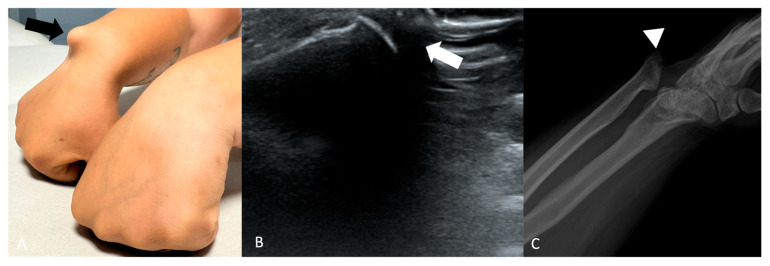
(**A**) Physical examination of a 27-year-old female shows a bump deformity on the medial side at the level of the radio-ulno-carpal joint (black arrow). (**B**) US shows the presence of abnormal bone (white arrow). (**C**) X-rays reveal a severe radioulnar-carpal joint deformity with dorsal ulnar metaepiphysis luxation in fracture outcomes (arrowhead).

A bony bump (Figure 5) may represent the consequence of a traumatic event (most common) or be due to underlying developmental anomalies. Hand and wrist injuries constitute 6.6% to 28.6% of all injuries in the musculoskeletal system. The most affected anatomical region of the upper extremity is the fingers (38.4%), followed by the wrists (15.2%) [26].

In case of a suspicious bony lesion, X-ray is the modality of choice and often allows the diagnosis without the need for other imaging modalities [27]. CT is particularly useful for assessing occult osseous abnormalities, such as undisplaced fractures, subtle subluxations, erosions, and osteolytic lesions [28]. On the other hand, MRI is the modality of choice for evaluating soft tissues—including the articular capsule, ligaments, fibrocartilage, tendons, and muscular attachments around the joint. It provides excellent visualization of capsuloligamentous injuries, from mild sprains to complete tears. MRI is also ideal for detecting occult fractures, bone marrow edema due to trauma, inflammation or infection, and early erosive changes [29].

**Figure 6:**
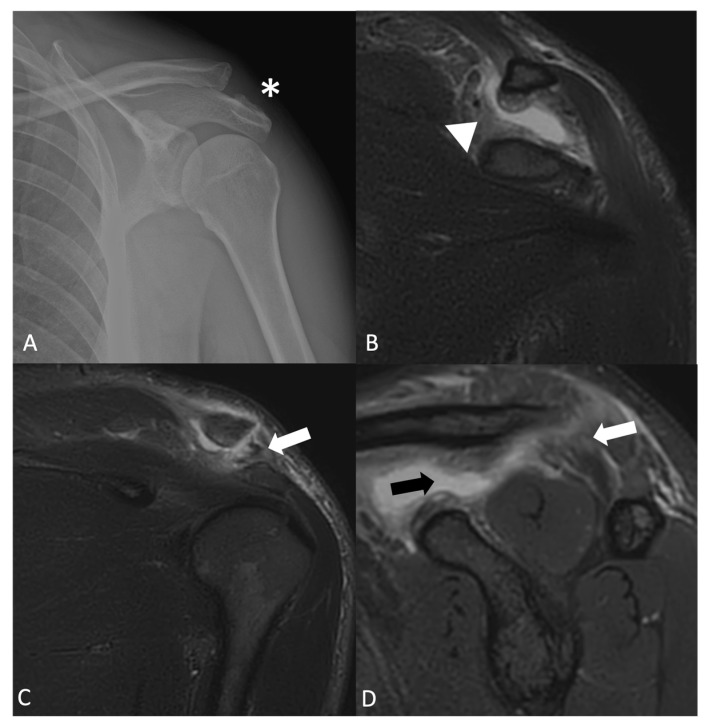
(**A**) AP X-ray, (**B**,**C**) coronal STIR, and (**D**) sagittal STIR in a patient with type III acromioclavicular dislocation according to the Rockwood classification. There is a rupture of both the acromioclavicular (white arrows) and coracoclavicular ligaments, trapezoid ligament rupture (black arrow), and conoid ligament rupture (arrowhead), causing complete dislocation of the joint (asterisk).

Acromioclavicular (AC) joint dislocation (Figure 6) may present as a bump above the AC joint [30].

**Figure 7:**
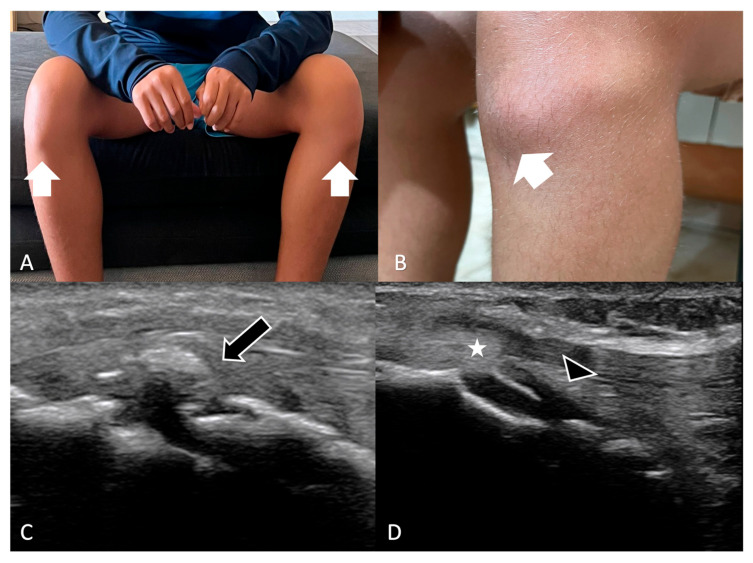
(**A**,**B**) Physical examination of a 12-year-old male reveals a bilateral deformity, presenting as bumps on the anterior aspect of both knees (white arrows), at the level of the tibial tuberosities. (**C**,**D**) Ultrasound examination shows irregularity and fragmentation of the anterior tibial apophysis growth nucleus (black arrows), thickening of the patellar tendon (white stars), and a small amount of fluid in the right intra-articular space (arrowhead).

The severity of AC joint dislocation is classified using the Rockwood classification system, with types I to VI representing increasing degrees of ligamentous disruption and joint instability [31]. Bony bumps are often seen in non-traumatic bones. Osgood-Schlatter (OS) disease (Figure 7) is a traction apophysitis due to the repetitive contraction of the quadriceps femoris muscle, which affects the patellar tendon, which in turn exerts traction against the tibial tubercle [32]. This condition is most common in children and adolescents who play sports, and it is more frequent in males than in females [33].

**Figure 8:**
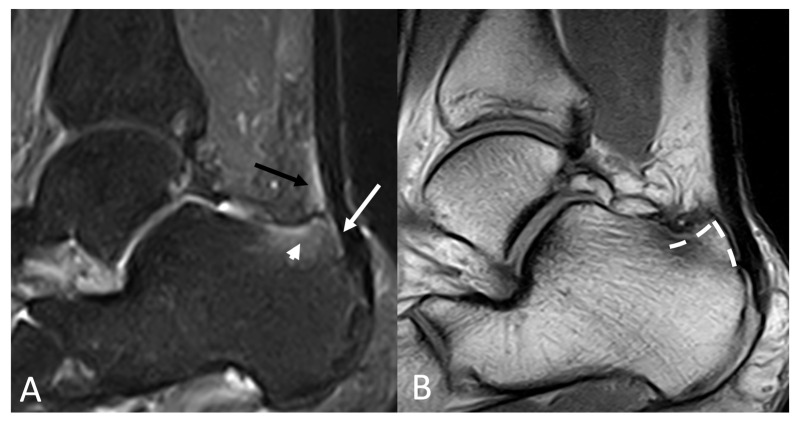
(**A**) Sagittal STIR and (**B**) sagittal T1-weighted of a 37-year-old male with pain and a bump at the level of calcaneal insertion of the Achille’s tendon. Bony edema of the calcaneus (arrowhead) and the area of impingement between the bone and the tendon (arrow). Retrocalcaneal bursitis is evidenced by increased fluid and inflammation (black arrow). A bony bump on the posterosuperior calcaneus (dashed line) is present, consistent with Haglund’s deformity.

Haglund Deformity (HD) (Figure 8) is a condition characterized by an enlargement in the form of exostosis in the posterosuperior region of the calcaneal bone. Haglund’s disease refers to the constellation of conditions caused by HD, including Achilles tendinitis, retrocalcaneal bursitis, supracalcaneal bursitis, and inflammation of the Kager fat pad [34].

In both OSD and Haglund’s Disease, X-ray allows the detection of bone deformity, but MRI provides a more sensitive and specific assessment for soft tissue evaluation, including tendons, bursae, and fat pads (e.g., tendon, fat pad, bursae) [34,35,36].

## 5. Solid Lesions

A solid lesion around a joint may represent a broad spectrum of possible differential diagnoses. The inguinal and axillary regions are also home to lymph node stations that can cause bumps. They can be increased in inflammatory pathologies but also in tumor pathologies with lymph node metastases: pelvic tumors (anus, bladder, cervix, endometrium, ovary, penis, prostate, rectum, testis, vagina, and vulva) usually metastasize first to regional inguinal lymph nodes [37]. Breast cancer represents the most frequent cause of enlarged axillary lymph nodes [38]; typical localization of lymphoma includes lateral cervical, axillary, and inguinal [39].

Ultrasound (US) is the modality of choice for evaluating lymph nodes and detecting potential malignancy [40,41,42,43].

Malignant features include:

Gray-scale criteria:

- Short axis to long axis ratio > 0.5
- Round shape
- Hypoechoic appearance
- Heterogeneous echotexture
- Loss or thinning of the central fatty hilum
- Eccentric cortical thickening
- Additional signs:
  ○ Microcalcifications
  ○ Necrosis (cystic or coagulative)
  ○ Ill-defined or irregular capsule margins

Color/Power Doppler findings:

- Peripheral or mixed vascular patterns
- High resistance flow (RI > 0.8, PI > 1.5)
- Aberrant vascular features:
  ○ Displaced or distorted hilar vessels
  ○ Subcapsular vascularity
  ○ Areas of non-perfusion
  ○ Non-tapering vessels

CT and MRI are useful for the detection of cancers underlying pathological lymph nodes.

Among benign tumors, Giant Cell Tumor of the Tendon Sheath (GCTTS) is the second most common mass of the hand after ganglion cysts [44]. It is a benign proliferative and inflammatory condition arising from the synovia of joints (in this case known as PVNS), bursae, or tendon sheaths. It commonly occurs in palmar locations but has a higher recurrence in extensor tendons [8]. GCTTS appears as a hypoechoic mass intimately related to the ring finger flexor tendon sheath at US examination; on dynamic examination, the GCTTS does not move with the tendon as it arises from the sheath rather than the tendon itself [45]. MRI shows a well-defined mass as well adjacent to or enveloping a tendon. Usually GCTTS shows low signal intensity in T2W imaging and blooming artifacts (a susceptibility artifact that appears as an exaggerated area of signal loss surrounding paramagnetic substances—such as hemosiderin, calcifications, or metal fragments—due to local magnetic field inhomogeneities) in Gradient Echo sequences due to repeated bleeding [45]. Synovial sarcoma (SS) is a malignancy characterized by mesenchymal spindle cell tumors with diverse epithelial differentiation [46]. This malignancy predominantly affects children and young adults (up to 32 y/o), with a higher incidence in males than in females [46,47]. Cases of primary intra-articular SS are extremely rare [48]. It typically presents as a large, deep-seated mass with rapid growth. It can be associated with knee effusions, which may or may not be accompanied by pain [46,49].

Plain radiography has limited sensitivity in detecting the mass unless it is significantly large and calcified [47]. MRI scans with and without contrast will show the lesion to be hypointense on T1-weighted images and hyperintense on T2-weighted images [10]. Large lesions may show multilobulation and heterogeneity, especially on T2-weighted images [46,49]. Smaller tumors (less than 5 cm) may have a more homogeneous signal and may be mistaken for benign lesions, especially if their margins are well defined. SS, on the other hand, shows intense but heterogeneous gadolinium enhancement, which helps to differentiate it from benign cystic lesions [49]. The presence of fluid levels with hemorrhage may produce the characteristic ‘bowl of grapes’ sign [50]. In addition, MRI shows bone infiltration in 21–28% of cases, although bone contact or erosion is even more common.

## 6. Conclusions

Periarticular soft tissue masses in the appendicular skeleton encompass a broad spectrum of lesions, including cystic, fatty, bony, inflammatory, and neoplastic origins. While the majority are benign, certain entities may behave aggressively or represent malignancy, making early and accurate diagnosis essential. A thorough clinical evaluation—considering lesion duration, size, growth rate, and associated symptoms—remains the first step in the diagnostic process. However, imaging plays a pivotal role in confirming the presence of a lesion, identifying its nature, guiding biopsy if necessary, and facilitating treatment planning. US remains the first-line modality for evaluating superficial masses, offering high spatial resolution and the ability to assess vascularity. MRI is invaluable in deeper or inconclusive cases, particularly for assessing lesion composition, extent, and relationship to adjacent structures. CT has a complementary role, particularly for calcified or osseous components and procedural planning. In many cases, imaging findings are sufficient to suggest a specific diagnosis and avoid unnecessary invasive procedures. Emerging technologies [51,52], such as artificial intelligence and deep learning, have shown promise in the automated classification of soft tissue lesions, potentially supporting radiologists in complex diagnostic scenarios. As highlighted by recent studies, GAN-based models like Pix2Pix may enhance lesion detection and classification, though clinical validation is still ongoing.

Ultimately, a structured, etiology-based approach combining clinical information and targeted imaging can improve diagnostic confidence, streamline management, and ensure appropriate patient care.

## Figures and Tables

**Table 1:** Overview of lesions that can present as bumps, including their incidence, typical age range, predominant gender, clinical signs, and natural evolution.

Lesion	Incidence/Prevalence	Typical Age Range (Years)	Predominant Gender	Clinical Signs	Natural Evolution
Ganglion Cysts	10–30% of wrist lesions	20–40	Female	Painless mass, mild discomfort with motion	Stable, sometimes spontaneous regression
Synovial Cysts	5–10% of articular lesions	30–50	Neutral	Local swelling, pain with activity	Slow growth, possible rupture
Liposarcoma	Rare (0.5–1% of soft tissue tumors)	50–70	Male	Deep mass, sometimes painful	Slow progression, malignant
Osteochondromas	1–2% of the general population	<20	Male	Bony projection, asymptomatic	Stable, rare malignant transformation
Pigmented Villonodular Synovitis	1.8/1,000,000	20–40	Neutral	Pain, stiffness, joint swelling	Locally aggressive progression
Giant Cell Tumors	15–20% of benign bone tumors	20–40	Female	Pain, swelling, reduced range of motion	Locally aggressive growth
Lipoma	2.1/100,000	40–60	Female	Soft, mobile, asymptomatic mass	Stable, benign
Bursitis	Common, varies with physical activity	All ages	Neutral	Local pain, swelling, warmth	Recurrent episodes

## Data Availability

No new data were created or analyzed in this study.
